# Supplementary material for: Between-day reliability of local and global muscle-tendon unit assessments in female athletes whilst standardising menstrual cycle phase
Source: PLoS One. 2025 Jun 3;20(6):e0306587. doi: 10.1371/journal.pone.0306587 (PMC12133186; doi:10.1371/journal.pone.0306587)
Supplement: S1 Table — (DOCX) [file pone.0306587.s001.docx]

S1 Table. Definitions and calculations of local muscle-tendon unit metrics.

| **Variable** | **Definition/calculation** |
| --- | --- |
| **Achilles’ tendon metrics** |  |
| Raw elongation (mm) | Maximum elongation of the AT measured from rest to MVC. |
| Passive elongation (mm) | The elongation of the AT due to joint rotation that occurs during maximal contractions. |
| Corrected elongation (mm) | Elongation of the AT from rest to MVC, corrected for the elongation due to passive joint rotation (i.e., raw elongation minus passive elongation). This variable is used for all strain and stiffness calculations. |
| Strain (%) | Elongation of the AT relative to resting length. |
| AT force (N) | Ankle plantar flexion moment (recorded by the dynamometer) divided by the AT moment arm. |
| AT*k* (N/m) | Stiffness – slope of the AT force-elongation curve from 0-100% (AT*k*_all_), 0-50% (AT*k*_low_), or 50-100% (AT*k*_high_) of MVC. |
| NAT*k* (N/strain) | Normalised stiffness – slope of the AT force-strain curve from 0-100% (NA*k*_all_), 0-50% (NAT*k*_low_), or 50-100% (NAT*k*_high_) of MVC. |
| AT*k*_index_ (N/strain) | Maximum tendon force divided by maximum tendon strain [19]. |
| **Single-joint isometric strength** |  |
| Ankle plantar flexion moment (N.m) | Average maximum plantar flexion moment recorded by the dynamometer across all maximal contractions. |
| Knee extension moment (N.m) | Average maximum knee extension moment recorded by the dynamometer across all maximal contractions. |
| AT, Achilles’ tendon; MVC, maximum voluntary contraction. | |
