# Supplementary material for: Between-day reliability of local and global muscle-tendon unit assessments in female athletes whilst standardising menstrual cycle phase
Source: PLoS One. 2025 Jun 3;20(6):e0306587. doi: 10.1371/journal.pone.0306587 (PMC12133186; doi:10.1371/journal.pone.0306587)
Supplement: S2 Table — (DOCX) [file pone.0306587.s002.docx]

S2 Table. Definitions and calculations of global muscle-tendon unit metrics.

| **Variable** | **Definition/calculation** |
| --- | --- |
| **Countermovement jump metrics** | |
| Jump height from take-off velocity (cm) | BioWare software is used to calculate the velocity of the centre of mass at take-off, and jump height is then calculated using the equation:  *d = v_f_^2^ / 2g*  *W*here *d* = jump height, *V_f_* = take-off velocity, and *g* = acceleration due to gravity. **Also used for squat jump.** |
| Jump height from flight time (cm) | *d = v_i_t + ½gt^2^*  Where *d* = jump height, *V_i_* = initial velocity, and *g* = acceleration due to gravity, and *t* = half of flight time. **Also used for squat jump and drop jump.** |
| RPD (W/s) | Slope of the power-time curve from the start of the concentric phase (first instant of positive velocity of the centre of mass) to the instant of peak power [43]. |
| RFD (N/s) | Slope of the force-time curve from the start of the braking phase (instant of maximum negative velocity of the centre of mass) to the instant of peak force [27]. |
| Positive impulse (N.s) | Integration of the vertical force-time curve from the instant that force rises above body weight (i.e., start of the braking phase) until take-off [30]. |
| Countermovement depth (cm) | Negative displacement of the centre of mass calculated via double integration (using trapezoid rule) of acceleration time data, which in turn was calculated by dividing force by body mass. |
| Vertical stiffness (N/m) | Peak force divided by countermovement depth [72]. |
| RSImod | Jump height (via flight time method) divided by time to take-off (defined as the time from onset to take-off. [73]). |
| **Squat jump metrics** | |
| RPD (W/s) | Slope of the power-time curve from onset to the instant of peak power. |
| RFD (N/s) | Slope of the force-time curve from onset to the instant of peak force. |
| RFD time bands (N/s) | Slope of the force-time curve from onset to the specified time point (i.e., 50, 100, or 250 ms). |
| Eccentric Utilisation Ratio | CMJ jump height / SJ jump height (via take-off velocity method [61]). |
| **Drop jump metrics** | |
| RSI | Jump height divided by ground contact time [74]. |
| **Isometric midthigh pull metrics** | |
| Impulse time bands (N.s) | Force curve integrated over specific time band (i.e., 50, 100 or 250 ms). |
| RFD time bands (N/s) | Slope of the force-time curve from onset to the specified time point (i.e., 50, 100, or 250 ms). |
| Dynamic Strength Index | CMJ peak force divided by IMTP peak force [62]. |
| RPD, rate of power development; RFD, rate of force development; RSImod, reactive strength index modified; RSI, reactive strength index. | |

72. Maloney SJ, Fletcher IM. Lower limb stiffness testing in athletic performance: a critical review. Sports Biomech. 2021;20(1):109–30.

73. Ebben WP, Petushek EJ. Using the reactive strength index modified to evaluate plyometric performance. J Strength Cond Res [Internet]. 2010;24(8):1983–7. Available from: www.nsca-jscr.org

74. Flanagan EP, Ebben WP, Jensen RL. Reliability of the reactive strength index and time to stabilization during depth jumps. J Strength Cond Res [Internet]. 2008;22(5):1677–82. Available from: www.nsca-jscr.org
