# Supplementary material for: Between-day reliability of local and global muscle-tendon unit assessments in female athletes whilst standardising menstrual cycle phase
Source: PLoS One. 2025 Jun 3;20(6):e0306587. doi: 10.1371/journal.pone.0306587 (PMC12133186; doi:10.1371/journal.pone.0306587)
Supplement: Attachment — Submitted filename: Response to Reviewers.docx [file pone.0306587.s005.docx]

07/08/24

*PLOS ONE*

Re: manuscript reference PONE-D-24-24939, ‘Between-day reliability of local and global muscle-tendon unit assessments in female athletes whilst controlling for menstrual cycle phase’.

Dear Dr Waugh,

We would like to thank yourself and the reviewers for your insightful comments and recommendations, as we feel that these have improved the quality of the manuscript. Please see uploaded the revised version of the manuscript (‘Revised Manuscript with Track Changes’), and our response to all individual comments below.

We hope that these revisions make our manuscript suitable for publication in *PLOS ONE*. We look forward to hearing from you.

Sincerely,

Scott Newbould

Carnegie School of Sport, Leeds Beckett University, United Kingdom

s.newbould@leedsbeckett.ac.uk

0113 812 8823

| **Reviewer #1 comments:** | **Author responses:** |
| --- | --- |
| General comments: This study provides new female dataset for various global performance measures (i.e., CMJ jump height, mid-thigh pulls metrics) and other quantifiable muscle-tendon characteristics, which expands the scientific understanding of test-retest reliability in these performance metrics in the given population. The introduction, discussion, and conclusion of this manuscript were well-written, so most of my review are on the technical sides (method). Please see below points raised: | We would like to thank the reviewer for taking the time to review our manuscript. Your constructive comments and recommendations led to some changes that we have made to the manuscript, which we feel have improved its overall quality. We have responded to all your individual comments (see below) and highlighted any manuscript changes in red text. We hope that the updated version is now of sufficient quality. |
| Line 132  The ultrasound image was collected at 15 Hz, which seems to be a lower frequency. Could the authors justify why 15 Hz was chosen in this study, and not 30 Hz or above? Did the authors compared similar isometric plantarflexions to other frame rates to ensure 15 Hz was sufficient? This could be one of the reasons why traditional stiffness results were less reliable in this study because there were less data points to fit a curve over a specified region to calculate these traditional stiffness values (and hence a less accurate curve was fitted). | Thank you for this comment. Whilst we agree that a sufficient sampling rate is crucial in many dynamic ultrasound applications, we do not feel it is relevant here. The reason for this is that we used a “point-to-point” method, where we located the myotendinous junction at rest, and then again during the isometric phase of each contraction (so, two discrete measurements as opposed to time-series tracking). We did this at each contraction intensity, and then found the slope of these data points to obtain stiffness. The lower sampling rate was chosen here to allow us to optimise the spatial resolution (image quality), which we felt was more important for these analyses. We have added clarity to the methods regarding this point (Lines 148-150). |
| Line 161-162  The authors mentioned “determined by the greatest flight time” to define the best three trials of the jump. However, throughout the method section, the authors did not explicitly define how jump height was calculated. The authors mentioned a software (Line 157) to make these calculations, but in my opinion, this was not particularly clear. Looking at Table 2, Table 3, and the supplementary file, the reported jump height included both the flight time-based calculation and the velocity-based calculation. It would be a good practice to explain clearly which method was used in this manuscript to determine test-retest reliability. Usually, velocity-based calculation is the “impulse method”, where the net vertical impulse of the jump during the entire ground contact phase was used to calculate change in COM velocity at take-off. Impulse method is generally more reliable than flight time method since it is not affected by landing posture. The authors addressed the limitation of flight time method in the discussion (Line 321), but please define the calculation method earlier in the method section. Also, please consider analysing velocity-derived jump height results if this has not been done. | Thank you for raising these points regarding the jump height calculations. We have added the flight time jump height calculation in the methods section for greater clarity (Line 176) and have also highlighted that the reliability of both jump height calculation methods is reported in this study (Line 178-180). |
| Line 163-164  This statement is mostly correct, but the term “isolate the lower body” might seem less accurate. This sentence might improve better if we change it to “remove the effect of arm swing to ensure jump contribution was predominantly lower limb driven” or something similar. The reason is, even without arm swing, the torso/trunk can still add to the final jump performance and therefore we cannot really isolate lower body, unless we perform jumps on a Smith machine that restricts trunk contribution. | Thank you for this comment. We agree with these points and have changed the manuscript accordingly (Lines 181-182). |
| Line 168-170  For the SJ protocol, the authors did not explain whether the “self-selected depth” for each participant was strictly controlled across different trials and/or different sessions. Unlike CMJ, the initial position of a SJ could change more easily than CMJ if not monitored carefully, making the SJ results a function of depth manipulation. Participants might vary their depth selection between trials or sessions, which might contribute to some variability in the data. The data reported in this study showed a high reliability for SJ jump height, but if this outcome was based on a variable squat depth, then our interpretation of SJ jump height would be more complicated. Please elaborate further on whether self-selected depth for SJ was controlled in the method section. If not controlled, it would be good to briefly mention in the discussion as a limitation. | Thank you for this comment. The self-selected SJ depth was used based on research by Petronijevic et al. (2018, citation #44 in the manuscript), which showed that self-selected SJ depth produced similar jump heights, but greater peak force and peak power than a standardised 90° knee angle. Furthermore, the self-selected depth was more reliable between sessions than the standardised depth in physically active participants with no prior SJ experience, which is important as the participants in the present study did not have prior SJ experience. Whilst we agree that participants might vary their depth between sessions and this might influence outcomes, we believe that this is a more ecologically valid approach that would be used by coaches, practitioners, and researchers. Additionally, CMJ depth could also differ between sessions but was not controlled, so both jumps were treated the same, in that the same instructions were given to participants in each session, but their depth was not controlled. A comment stating the depths were not controlled has been added (Line 349-351). |
| Line 261-276  This paragraph is well-written. One extra point to consider is that traditional stiffness calculation can (not always) involve fitting a curve to the raw data, and therefore the stiffness values can be affected hugely by the shape of the fitted curve. On the other hand, the method adopted in this study divided the maximal AT force by the maximal strain, which did not require curve fitting and reduced the possibility of errors associated with curve fitting. This is just a comment, so the authors can decide whether to agree with this comment or not. | Thank you for this comment, as it raises important points regarding stiffness calculations that must be considered. We agree that curve fitting can influence the stiffness values for traditional stiffness metrics, but not for AT*k*_index­_. We used a linear slope for the traditional stiffness metrics as this typically provided the best fit for the data and is also very commonly used in the literature. |

| **Reviewer #2 comments:** | **Author responses:** |
| --- | --- |
| General comments: The current study aimed to test-retest reliability of local and global MTU function assessments during the early follicular phase of the menstrual cycle. The topic is highly valuable to the scientific community as research with females is largely neglected in MTU both in cross-sectional but even more specifically in interventional studies. The manuscript is generally well-written, but includes several points to improve prior being fit for publication. Please see my comments / suggestions below. | We would like to thank the reviewer for taking the time to review our manuscript. We believe the manuscript quality has been improved after responding to your comments and suggestions. Please see below our response to all your individual comments, and we have highlighted any manuscript changes in red text. We hope that the updated version is now of sufficient quality. |
| Title and the whole manuscript (starting for instance L48): Terms “local and global MTU function” are not quite correct. Function does not equal mechanical properties. Mechanical properties like tendon strain or stiffness are quantities that describe material characteristics, which can affect function. In the case of the tendon, its function is to transmit forces and store/release energy and this is influenced by its mechanical properties. I would suggest to use “local and global MTU assessment“, then you are on a correct side. Double check the whole manuscript if the term “function” is used correctly. | Thank you for this comment; we agree with this perspective and have edited the manuscript to reflect this. |
| L58-L60: Biological variation in what exactly? | Thank you for this comment. The biological variation we refer to here refers to the differences in hormone concentrations that exist between the sexes, as highlighted in the next sentence. To clarify this, we have changed the wording from ‘additionally’ to ‘specifically’ at the start of the next sentence. (Line 60). |
| L73: Change “jump” to “jumping” | Thank you, content amended accordingly (Line 73). |
| L76-L81: It is quite hard to compare those studies due to the methodological differences (i.e. different joint configurations, accounting for inevitable joint changes to measured elongation, the region used for calculating tendon stiffness, differences in loading protocol), which explains largely the variation in the reliability. Therefore I would be careful with the statement of “mixed findings” because the least controlled and less favorable conditions tend to lead to a lower reliability / higher variability. Pooling these results in one pot is not a wise and legitimate way to interpret the findings from these studies. I think the authors should recognize the reasons why this cannot be done. | Thank you for this comment. We agree with the points raised in your comment and have included consideration of these aspects in the paragraph. In light of this, we have also removed the phrase “mixed findings” as we agree that it is not an appropriate phrase to use when studies have used different methodologies (Lines 77-82). |
| L99: “..., whilst controlling for menstrual cycle phase.” is a weird way to say it, because you are not really controlling it rather than performing the measurements within a specific phase of the menstrual cycle. | Thank you for this comment. We agree and have amended the manuscript to use the term ‘standardised’ rather than ‘controlled’ throughout. |
| L123: “of AT mechanical function” is not correct. Change it to “mechanical properties”. | Thank you, content amended (Line 126). |
| L128: I think a brief explanation of the Barfod et al. 2015 method using US to measure tendon length would be helpful for the readers, because it is not really a conventional measure across different studies and research groups. Mention that it takes into account the curvature of the AT. | Thank you for this comment, we have added a brief description of this technique (Lines 131-136). |
| L144: Did you also account for the axis misalignment between the ankle joint centre and the dynamometer’s axis of rotation? If not this can affect directly the plantarflexion MVCs. Considering the inevitable ankle joint rotation seemed to be more than 10deg, the peak joint moments would have been produced in a less optimal joint configuration. | Thank you for this comment, which highlights an important consideration for dynamometer testing. In this study we did not account for the misalignment of these axes, which we understand would induce some degree of error to the plantar flexion MVC values. However, as can be seen by the reliability statistics in Table 1, the magnitude of ankle joint rotation was not different between sessions. Therefore, although there may be error in the plantar flexion MVCs, assuming consistent MVC scores, this error should be systematic across testing sessions and therefore not influence the reliability results. Discussion of this has been added to the methods section (Lines 152-156). |
| L149: “To investigate the reliability of the manual measurement of AT elongation, a random sample of 10 maximal contractions were selected from the whole dataset.” I do not see the reasoning for not including all the maximal contractions from all the participants for the reliability analysis. This could potentially artificially increase the intra- and inter-rater reliability. | Thank you for this comment. The reliability of the manual measurement of AT elongation was not central to this study; we have focused on the reliability of the overall method and of different stiffness calculation methods and force ranges. However, as the intra- and inter-rater reliability provide useful information regarding the contribution of error to the overall stiffness outcomes, we have included these measurements for robustness. Of the previous research cited in the introduction that reports the reliability of AT elongation/stiffness, many used similar sample sizes to the present study for their reliability analyses. For example, Kubo et al., 2002, Hansen et al., 2003, Burgess et al., 2007, Fouré et al., 2010, Rogers et al., 2017, and Hunter et al., 2022 used 8, 11, 13, 8, 11, and 11 participants, respectively, for an average of 10. We therefore believe that our *n* of 10 for the intra- and inter-rater reliability analyses and 17 participants for the full analysis is sufficient. Finally, these data provide reliability statistics specifically for the participants in the study, whereas other research has at times relied on pilot or previous work and applied those findings to a different sample of participants. |
| L126-L154: Did I miss it or you have forgotten to describe how you measured the absolute tendon stiffness and normalized tendon stiffness? Because in the results you are demonstrating the ICC values etc. Which region of the force-elongation relationship was used for calculating the slope etc. | Thank you for this comment. The definitions and calculations for all metrics are provided in the Supplementary material to maintain brevity and conciseness in the manuscript. We have included another reference to the Supplementary tables after the AT mechanical properties testing procedures to make this clearer to readers (Lines 150-151). |
| L177-L190: Did you take into account the inevitable axis misalignment between the isokinetic device and the knee joint centre? If not this can directly affect your outcomes and needs to be mentioned here and in the limitations. | The magnitude of axis misalignment was not measured for knee extension, but as above with the plantar flexion comment, knee extension moments were similar between sessions, and so again the magnitude of error would be similar between sessions. |
| L183-L186: How were the joint configurations determined? Motion capture? During the movement there are definitely inevitable changes in the joint angles. Does and how much could this affect the resultant measured peak GRF. Only the left leg stood on the force plate? I think needs to be mentioned. And if not, then why? | Thank you for this comment. To tackle the latter aspect first, the IMTP was performed bilaterally, not unilaterally. The methods content has been updated to clarify this (Line 197). Bilateral IMTP and vertical jumps were used instead of unilateral as these are arguably more commonly used in practice, and as such, providing reliability data for these is of greater benefit to the female athlete scientific community.  Joint configurations were determined with a goniometer before the trials. During the trial there will be inevitable joint angle changes, despite participants receiving coaching cues to reduce this. Although this will impact the resultant GRF measured, we believe that these data provide an ecologically valid measure of the IMTP reliability, as correction for joint angle changes is not often performed in applied practice or research. |
| Table 2, 3, 4 and 5: You have not described how you calculated most of the parameters, just referred to a previous study. Surely some of them need explaining. | Thank you for this comment. The definitions and calculations for each variable are provided in the supplementary material S1 and S2 tables. We have added this information to the table legends to make this clearer to readers. |
| L259 and L373: “blunt instruments” I would refrain from using this term in the manuscript. | These have been removed and replaced with “may lack the precision to…” (Lines 276-277 and 394). |
| L438: In the reference Robshaw DC, you are missing the full information about this PhD Thesis. | Thank you for highlighting this. The full information is now included. |

| **Journal requirement** | **Author responses** |
| --- | --- |
| 1. Please ensure that your manuscript meets PLOS ONE's style requirements, including those for file naming. The PLOS ONE style templates can be found at  https://journals.plos.org/plosone/s/file?id=wjVg/PLOSOne_formatting_sample_main_body.pdf and  https://journals.plos.org/plosone/s/file?id=ba62/PLOSOne_formatting_sample_title_authors_affiliations.pdf | Thank you for this comment. We have reviewed the style and file naming requirements and believe the manuscript now adheres to these. |
| 2. We note that you have indicated that there are restrictions to data sharing for this study. PLOS only allows data to be available upon request if there are legal or ethical restrictions on sharing data publicly. For more information on unacceptable data access restrictions, please see http://journals.plos.org/plosone/s/data-availability#loc-unacceptable-data-access-restrictions.  Before we proceed with your manuscript, please address the following prompts:  a) If there are ethical or legal restrictions on sharing a de-identified data set, please explain them in detail (e.g., data contain potentially identifying or sensitive patient information, data are owned by a third-party organization, etc.) and who has imposed them (e.g., a Research Ethics Committee or Institutional Review Board, etc.). Please also provide contact information for a data access committee, ethics committee, or other institutional body to which data requests may be sent.  b) If there are no restrictions, please upload the minimal anonymized data set necessary to replicate your study findings to a stable, public repository and provide us with the relevant URLs, DOIs, or accession numbers. For a list of recommended repositories, please see  https://journals.plos.org/plosone/s/recommended-repositories. You also have the option of uploading the data as Supporting Information files, but we would recommend depositing data directly to a data repository if possible.  We will update your Data Availability statement on your behalf to reflect the information you provide. | Thank you for this comment. We have uploaded the data to a recommended repository; figshare, with the DOI https://doi.org/10.6084/m9.figshare.26477206.v1. |
| 3. When completing the data availability statement of the submission form, you indicated that you will make your data available on acceptance. We strongly recommend all authors decide on a data sharing plan before acceptance, as the process can be lengthy and hold up publication timelines. Please note that, though access restrictions are acceptable now, your entire data will need to be made freely accessible if your manuscript is accepted for publication. This policy applies to all data except where public deposition would breach compliance with the protocol approved by your research ethics board. If you are unable to adhere to our open data policy, please kindly revise your statement to explain your reasoning and we will seek the editor's input on an exemption. Please be assured that, once you have provided your new statement, the assessment of your exemption will not hold up the peer review process. | As above, the data is now freely available to access on figshare. |
| 4. Please review your reference list to ensure that it is complete and correct. If you have cited papers that have been retracted, please include the rationale for doing so in the manuscript text, or remove these references and replace them with relevant current references. Any changes to the reference list should be mentioned in the rebuttal letter that accompanies your revised manuscript. If you need to cite a retracted article, indicate the article’s retracted status in the References list and also include a citation and full reference for the retraction notice. | Thank you for highlighting this. The citation of Robshaw (2021) has been updated to include all relevant information. |
