# Supplementary material for: Between-day reliability of local and global muscle-tendon unit assessments in female athletes whilst standardising menstrual cycle phase
Source: PLoS One. 2025 Jun 3;20(6):e0306587. doi: 10.1371/journal.pone.0306587 (PMC12133186; doi:10.1371/journal.pone.0306587)
Supplement: Attachment — Submitted filename: reviewer comment.docx [file pone.0306587.s003.docx]

This study provides new female dataset for various global performance measures (i.e., CMJ jump height, mid-thigh pulls metrics) and other quantifiable muscle-tendon characteristics, which expands the scientific understanding of test-retest reliability in these performance metrics in the given population. The introduction, discussion, and conclusion of this manuscript were well-written, so most of my review are on the technical sides (method). Please see below points raised:

Line 132

The ultrasound image was collected at 15 Hz, which seems to be a lower frequency. Could the authors justify why 15 Hz was chosen in this study, and not 30 Hz or above ? Did the authors compared similar isometric plantarflexions to other frame rates to ensure 15 Hz was sufficient? This could be one of the reasons why traditional stiffness results were less reliable in this study because there were less data points to fit a curve over a specified region to calculate these traditional stiffness values (and hence a less accurate curve was fitted).

Line 161-162

The authors mentioned “determined by the greatest flight time” to define the best three trials of the jump. However, throughout the method section, the authors did not explicitly define how jump height was calculated. The authors mentioned a software (Line 157) to make these calculations, but in my opinion, this was not particularly clear. Looking at Table 2, Table 3, and the supplementary file, the reported jump height included both the flight time-based calculation and the velocity-based calculation. It would be a good practice to explain clearly **which method** was used in this manuscript to determine test-retest reliability. Usually, velocity-based calculation is the “impulse method”, where the net vertical impulse of the jump during the entire ground contact phase was used to calculate change in COM velocity at take-off. Impulse method is generally more reliable than flight time method since it is not affected by landing posture. The authors addressed the limitation of flight time method in the discussion (Line 321), but please define the calculation method earlier in the method section. Also, please consider analysing velocity-derived jump height results if this has not been done.

Line 163-164

This statement is mostly correct, but the term “isolate the lower body” might seem less accurate. This sentence might improve better if we change it to “remove the effect of arm swing to ensure jump contribution was predominantly lower limb driven” or something similar. The reason is, even without arm swing, the torso/trunk can still add to the final jump performance and therefore we cannot really isolate lower body, unless we perform jumps on a Smith machine that restricts trunk contribution.

Line 168-170

For the SJ protocol, the authors did not explain whether the “self-selected depth” for each participant was strictly controlled across different trials and/or different sessions. Unlike CMJ, the initial position of a SJ could change more easily than CMJ if not monitored carefully, making the SJ results a function of depth manipulation. Participants might vary their depth selection between trials or sessions, which might contribute to some variability in the data. The data reported in this study showed a high reliability for SJ jump height, but if this outcome was based on a variable squat depth, then our interpretation of SJ jump height would be more complicated. Please elaborate further on whether self-selected depth for SJ was controlled in the method section. If not controlled, it would be good to briefly mention in the discussion as a limitation.

Line 261-276

This paragraph is well-written. One extra point to consider is that traditional stiffness calculation can (not always) involve fitting a curve to the raw data, and therefore the stiffness values can be affected hugely by the shape of the fitted curve. On the other hand, the method adopted in this study divided the maximal AT force by the maximal strain, which did not require curve fitting and reduced the possibility of errors associated with curve fitting. This is just a comment, so the authors can decide whether to agree with this comment or not.
